# How and why kinetics, thermodynamics, and chemistry induce the logic of biological evolution

**DOI:** 10.3762/bjoc.13.66

**Published:** 2017-04-07

**Authors:** Addy Pross, Robert Pascal

**Affiliations:** 1 Department of Chemistry, Ben Gurion University of the Negev, Be’er Sheva 84105, Israel; 2 NYU Shanghai, 1555 Century Avenue, Pudong New Area, Shanghai, 200122, China,; 3 Institut des Biomolécules Max Mousseron, UMR5247 CNRS-University of Montpellier-ENSCM, CC17006, Place E. Bataillon, Montpellier F-34095, France

**Keywords:** dynamic kinetic stability, kinetic control, origins of life, self-organisation

## Abstract

Thermodynamic stability, as expressed by the Second Law, generally constitutes the driving force for chemical assembly processes. Yet, somehow, within the living world most self-organisation processes appear to challenge this fundamental rule. Even though the Second Law remains an inescapable constraint, under energy-fuelled, far-from-equilibrium conditions, populations of chemical systems capable of exponential growth can manifest another kind of stability, dynamic kinetic stability (DKS). It is this stability kind based on time/persistence, rather than on free energy, that offers a basis for understanding the evolutionary process. Furthermore, a threshold distance from equilibrium, leading to irreversibility in the reproduction cycle, is needed to switch the directive for evolution from thermodynamic to DKS. The present report develops these lines of thought and argues against the validity of a thermodynamic approach in which the maximisation of the rate of energy dissipation/entropy production is considered to direct the evolutionary process. More generally, our analysis reaffirms the predominant role of kinetics in the self-organisation of life, which, in turn, allows an assessment of semi-quantitative constraints on systems and environments from which life could evolve.

## Introduction

Although it is mostly understood in historic terms, the origin of life constitutes a well-established field of research in chemical science [1] even though identifying the actual pathway by which life emerged on the early Earth will likely forever remain out of reach. The corresponding historical events left no record owing to the instability of the chemical components of the first living organisms and the tool of phylogenetic analysis is also limited, due to what might be called a horizon of knowledge, one which has been associated with the theoretical concept of the last common ancestor [2]. Current living organisms on Earth, in their extraordinary diversity, are unable to provide information on preceding stages of evolution that reach back beyond that horizon. And since the last common ancestor corresponds to an organism endowed with most of the essential functions present in current cells, phylogenetic studies are of little help when tackling the very early stages of life. The only alternative possibility is then to consider prebiotically available chemical pathways, as well as the constraints for chemical self-organisation, and to attempt to answer two questions: (1) Is there a driving force towards self-organisation of the kind observed in the living state? (2) If so, by what mechanistic means can a chemical system self-organise to yield the living state, consistent with the constraints of the Second Law. These considerations infer that an overall spontaneous decrease in entropy is statistically highly unlikely, and for macroscopic systems, effectively impossible. Accordingly, the emergence of life as the result of a single unlikely event is highly improbable [3–5]. Any alternative approach worthy of scientific investigation would therefore require the existence of a driving force for self-organisation, one necessarily associated with the production of entropy in the environment. The identification of such a driving force would make it possible to determine the parameters influencing change, even though no historical information regarding its early expression is available. Furthermore, identification of that driving force could serve as a logical bridge connecting the general rules governing change in the universe with Darwin's theory of evolution. Indeed, analysis of the thermodynamics of the processes considered to underlie life’s emergence might assist in closing the conceptual gap that continues to separate the physical and life sciences [6–7]. But does this mean that the history of the early evolution of life could be deterministically reconstituted through identification of life’s driving forces? The answer is certainly negative. The number of available chemical degrees of freedom is such that an almost infinite number of paths could potentially have been followed, so contingent events, historical by necessity, would also have had to play a cardinal role in determining the specific pathway that life processes happened to have taken. This statement does not preclude the possible occurrence of chemically predisposed pathways that could induce the selective formation of limited sets of building blocks potentially favourable toward that transition [8–9].

Much work has previously been devoted to the physicochemical characterisation of life. These attempts can be divided into two major approaches. Authors favouring a thermodynamic approach have emphasised the fact that life corresponds to dissipative processes taking place far from equilibrium [10], thereby explaining how self-organisation can arise without violation of the Second Law [11]. On the other hand, experimental molecular evolution [12] as well as theoretical developments [13–14] have supported a kinetically based view. Taking that kinetic approach, the concept of natural selection was able to be extended beyond biology so as to be applicable at the molecular level. Both views progressed separately in a context dominated by the RNA world hypothesis, though that hypothesis failed to eliminate the fundamental dilemma, as it led to conflicting so-called genetic and metabolic approaches to the origin of life [15].

Actually, as early as 1922, Lotka’s pioneering work, through two consecutive articles published in the same issue of PNAS and entitled “Contribution to the energetics of evolution” [16] and “Natural selection as a physical principle” [17], respectively, considered both approaches to the problem in order to account for the specificity of life (though the issue of the origin was not mentioned). This simple fact demonstrates how intimately bound he considered the metabolic and genetic features of life to be. Any physicochemical description of the origin of life that seeks to identify the physical principles responsible for life’s emergence should therefore take both considerations into account. Indeed, we believe it is through such a dual approach that a theoretical framework for describing the origin of life can be established, one able to help identify the driving forces responsible for self-organisation, as well as identify possible conditions able to support life’s emergence and early development. Thus the present work, extending ideas described in some detail in a series of earlier publications, is aimed at outlining the central features of a physicochemical approach to the origin of life, one which emphasises its kinetic character – how the evolutionary process from its outset is kinetically rather than thermodynamically determined, and provides new information in support of that view.

## Results and Discussion

### From thermodynamic self-assembly to kinetic self-assembly

**Figure 1:**
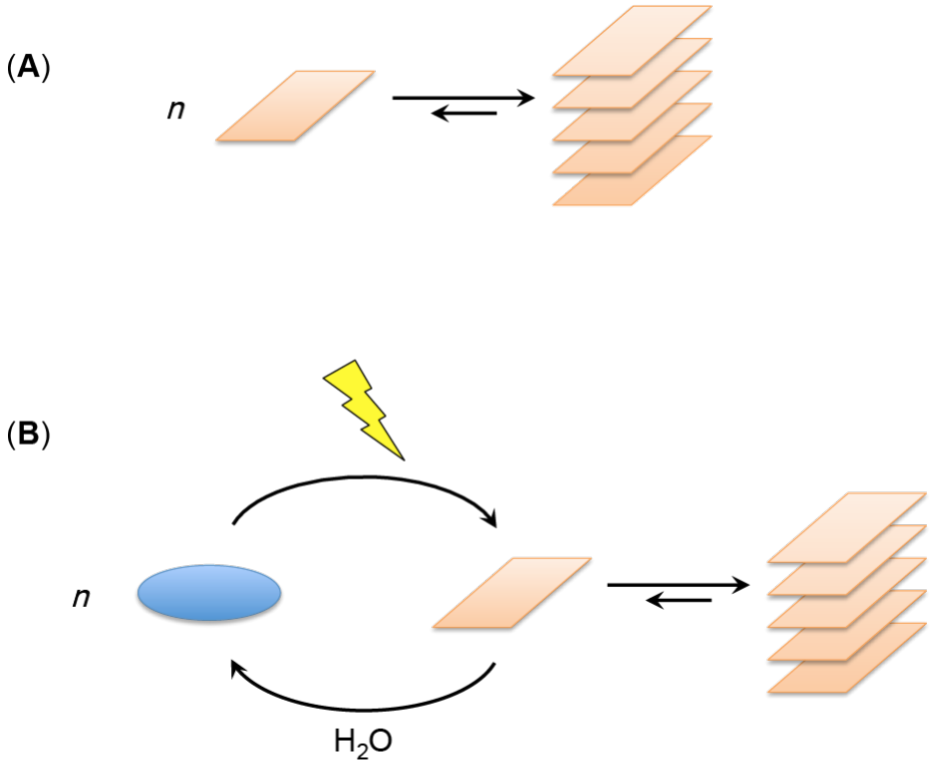
Self-assembly. (A) Macromolecular structures or patterns can form as the result of binding energy being released through the interaction of units which compensates for the decrease in entropy associated with self-organisation. (B) An example of dissipative self-assembly of reactants unable to react in the ground state but which can be activated to yield unstable reactive units (e.g., susceptible to hydrolysis). The supramolecular structure is dynamically stable as long as the system is held far from equilibrium through the energy-fuelled supply of reactive units.

Organised supramolecular structures are commonly formed when favourable interactions lead to the assembly of different components [18]. The release of chemical binding energy, i.e., the realisation of potential energy by dissipation of heat into the environment, compensates for the decrease in entropy associated with the loss of degrees of freedom of the individual chemical components. The increase in thermodynamic stability therefore constitutes the driving force for self-organisation, as required by the Second Law (Figure 1A).

With regard to living organisms, the situation is more complex. On the one hand, association processes directly driven by the Second Law are common in living organisms (e.g., protein folding, the assembly of protein sub-units through molecular recognition, assembly of nucleic acid duplexes as well as that of phospholipids to form a bilayer membrane). On the other hand, even though the Second Law must always remain an inescapable constraint, a simple drift towards the equilibrium state is not sufficient to account for the evolutionary changes of life. More elaborate processes, in particular that of increasing complexity, are clearly involved. As an example of a higher degree of complexity, out-of-equilibrium self-assembly can be observed when reactants that have no affinity for self-assembly in themselves, can be converted upon activation into transient species which can interact, leading to macromolecular structures or patterns [18]. The kinetic stability of the organised structures in those cases is associated with energy dissipation from an activating agent able to convert some reactant into transient species able to undergo intermolecular association (Figure 1B). These structures therefore result from dissipative self-assembly for which fascinating examples have been provided in the recent literature [19–21]. In biology, one of the most typical examples of this kind of assembly processes can be found in the dynamics of the cytoskeleton. However, even if these processes can explain some particular features of living organisms, they are not sufficient by themselves to constitute a driving force towards the self-organisation of living systems and to explain how life itself could have emerged and evolved.

### Life as a dissipative process emerging far from equilibrium

It has recently been claimed that thermodynamics could drive the self-organisation of life through an increase of energy dissipation rates [22–23], or, alternatively, in a continuing focus on the energy facet, that the evolutionary process takes place such that the total energy flux through the system is increased [16]. In yet another thermodynamic variant, it has also been suggested that the process leads to a maximisation of energy intensity [24]. Though Lotka introduced the maximisation concept, he was explicitly reluctant in making this proposal an absolute principle. This cautious approach has not been shared in more recent studies, in which a so-called “maximum entropy production” (MEP) principle, applicable within different fields of physical, biological and environmental sciences, has been introduced as an extension of the Second Law (see for example: [25–29]). That principle has also been seen as relevant when considering the origin and evolution of life problem (see for example: [30–35]). According to that proposal, a system that is held in a far from equilibrium situation should evolve towards an increase of energy dissipation and along a pathway in which the rate of dissipation, and thus of entropy production, is maximised. This approach, as well as closely related ones [22–23,36], expresses the view that the life phenomenon could therefore just be a consequence of a tendency of systems to maximise the dissipation of energy so that more complex systems, ones able to act as more effective dissipators, would be selected for. Also, it should be emphasised that though the MEP principle refers to the rate of entropy production, the basis for the “maximum entropy production” principle remains fundamentally thermodynamic, not kinetic, and, as will be discussed, that thermodynamic approach is opposed by more recent theoretical considerations, as expressed by Ross et al. [37] and our own analyses, described subsequently.

**Figure 2:**
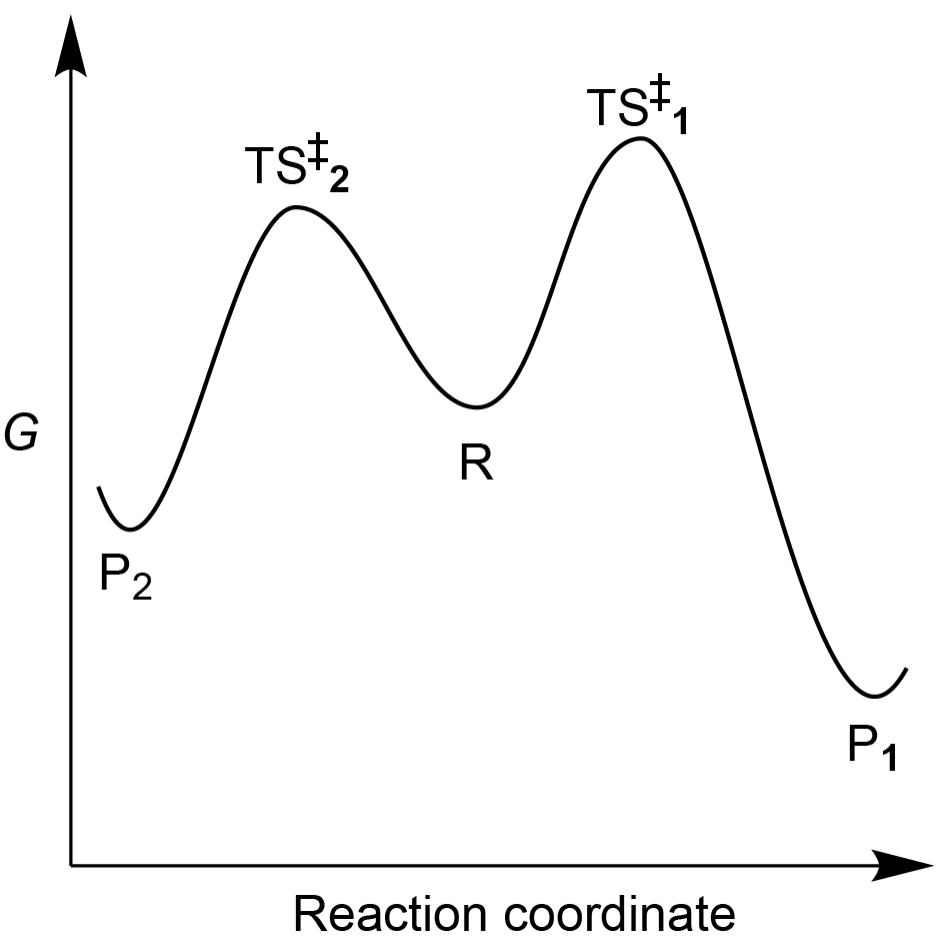
Kinetic control. In many chemical reactions leading to different products, the final composition is determined by the height of the kinetic barriers corresponding to transition states (**TS^‡^_1_** and **TS^‡^_2_**) rather than by the relative free energies of reactant (**R**) and products (**P_1_** and **P_2_**). Under kinetic control, **P_2_** would be favoured over **P_1_**.

More detailed views on the role of thermodynamics in biology have been critical of the position that natural selection expresses the drive towards maximum entropy production/energy dissipation/flux of reactants, and have proposed a less simplistic relationship that takes into account the self-reproducing property of living entities [17,38–42]. That approach toward living organisms [1,4,6–7,43–54] also favours a kinetic approach rather than a thermodynamic one, since there is no direct relationship between Gibbs free energy of reactions and kinetic barriers [37]. Indeed, the most significant flaw in attempts to derive natural selection from thermodynamics is that the kinetic behaviour of complex systems can hardly be deduced from data governing free energy minima, data which ignores the free energy barrier heights separating reactants and products. Organic chemists are fully cognisant of the fact that kinetic barriers cannot usually be deduced from thermodynamic data. Indeed, there are many examples in which product formation is controlled by kinetics (reactions under kinetic control, corresponding to the situation in Figure 2), rather than by thermodynamic stabilities. In fact the presence of kinetic barriers is actually a requirement for the system to be held far from equilibrium [43–44] so that life can only evolve from systems tightly bound, typically through covalent bonds [55–56]. Activated chemical species involved in these systems would not rapidly evolve at low temperature allowing the selection of efficient catalytic processes [50]. This observation therefore can explain the emergence of processes that lead to increased rates of transformation, and therefore energy dissipation. Thus one might say that the driving force for the emergence of life is related to the circumvention of kinetic barriers [42–43] rather than a consequence of the Second Law acting on a system held far from equilibrium.

Based on detailed physicochemical analyses, the idea of a MEP principle has indeed attracted criticism [37,57] and specific examples that are inconsistent with a thermodynamic directive have been discussed [37]. In addition, the expectation that biological systems would evolve towards systems exhibiting maximum entropy production is contradicted by the high yield that is observed in the conversion of nutrients into cell components, as for instance during glucose metabolism. In this case entropy production only slightly exceeds the minimum required by the Second Law indicating that the cell has evolved to minimise entropy production [58], not to maximise it. That observation in itself clearly shows that the production of cellular components is more important to the cell than the dissipation of energy. Indeed, in further support of a kinetic approach to evolution we have proposed [51] that the driving force for evolution can be identified as an expression of a persistence principle – a tendency of systems to evolve towards states in which their ability to change is reduced until they eventually reach a stable/persistent state in which no further change takes place. Though that idea is usually expressed in isolated systems as the Second Law, it can manifest itself as a trend toward greater DKS for populations able to reproduce themselves under favourable conditions. Actually, the probabilistic drive towards equilibrium expressed by the Second Law is replaced by a new one based on the mathematics of exponential growth for systems able to reproduce themselves in far from equilibrium situations [51–54]. In sum, as Ross et al. have pointedly noted: “predictions based on MEP-like principles should not be considered scientifically founded” [37]. Indeed, to strengthen that conclusion we now offer a kinetic simulation for a self-reproducing chemical system which further questions the generality of the MEP principle reaffirming the importance of kinetic considerations for such systems.

**Figure 3:**
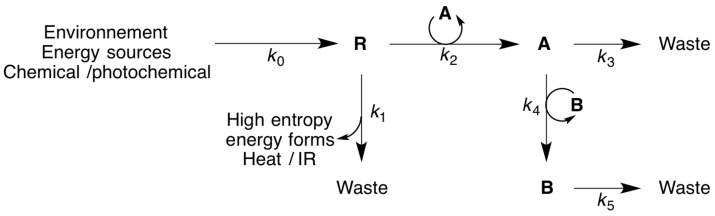
Evolution of an autocatalytic network involving a parasite. **R**: resource; **A**: autocatalyst; **B**: predator autocatalyst.

**Figure 4:**
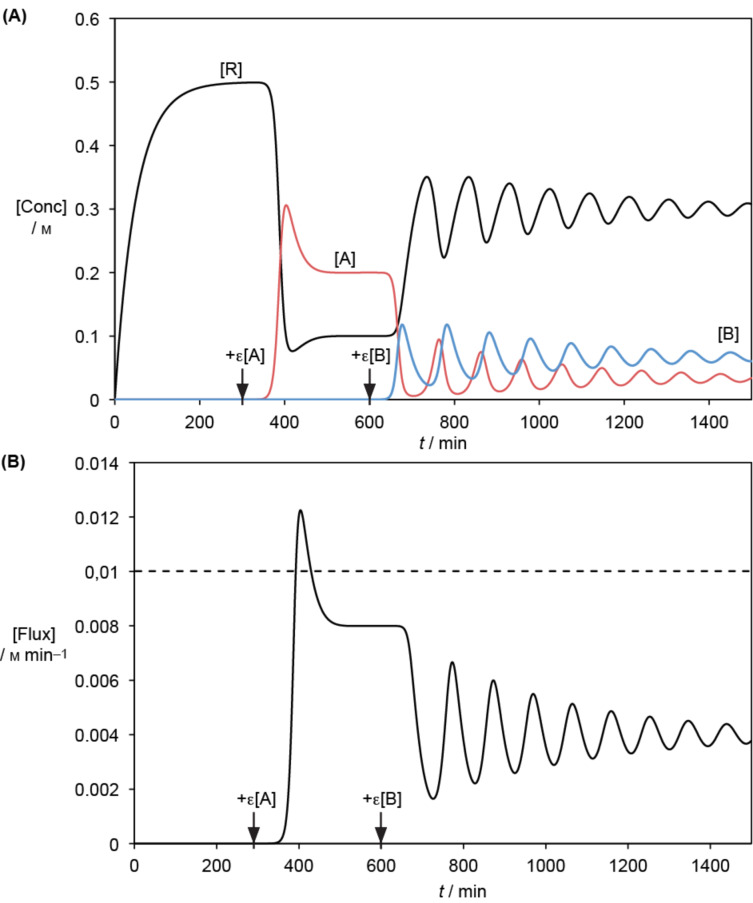
Numerical simulation of the system of Figure 3 (*k*_0_ = 0.01 M min^−1^, *k*_1_ = 0.02 min^−1^, *k*_2_ = 0.4 M^−1^ min^−1^, *k*_3_ = 0.04 min^−1^, *k*_4_ = 1.2 M^−1^ min^−1^ and *k*_5_ = 0.04 min^−1^). (A) Evolution of the concentrations of resource (**R**), autocatalyst (**A**) and predator (**B**) species; (B) flux of product formation through the autocatalytic system from **A** and **B**. The initial concentrations [R] = [A] = [B] = 0 were selected. After [R] approaches a steady state ([R]_*1_ = *k*_0_/*k*_1_ = 0.5 M), at 300 min 10^−6^ M **A** is added leading to a new steady state ([R]_*2_ = *k*_3_/*k*_2_ = 0.1 M and [A]_*2_ = (*k*_0_ − *k*_1_ × *k*_3_/*k*_2_)/*k*_3_ = 0.2 M). A new regime is initiated by the addition of 10^−6^ M **B** at 600 min (steady state [A]_*3_ = *k*_5_/*k*_4_ = 0.033 M and [R]_*3_ = *k*_0_/(*k*_1_ + *k*_2_ *k*_5_/*k*_4_) = 0.3 M). Simulation results were not changed using a twice-shorter interval of time between iterations (0.5 min instead of 1 min).

Consider a chemical system in which a chemically activated reagent (resource **R**) is produced transiently (Figure 3). After a delay required for equilibration, a minute concentration of an autocatalyst **A**, growing at the expense of this resource (Figure 3), is added to the system (see Figure 4). Given numerical simulation of rate constants, *k*_2_ and *k*_3_, for which the autocatalyst is viable (see Supporting Information File 1), the system is found to evolve irreversibly in the direction of increasing reactant flux corresponding to the autocatalytic dissipative process (catalysed by **A**) compared to its initial value. Changes in both the kinetic stability and reactant flux (reflecting entropy production through the dissipative process associated with autocatalytic step, *k*_2_) take place until a new steady state is achieved (after a transient peak).

Consider now the case in which a minute concentration of a parasite autocatalyst **B** formed from **A** and behaving as a predator, is introduced into the system. Surprisingly, for certain sets of rate constants (see Supporting Information File 1), the parasite can persist, but its incorporation into the system leads to a decrease in the overall reactant flux towards dissipation associated with autocatalytic step, *k*_2_. Note also that once the system with the parasite becomes stable (depending on the ratio of rate constants *k*_4_/*k*_5_; see Supporting Information File 1), it does not revert to the preceding state. The key point however: instead of the system evolving towards an increase in energy dissipation, parasite addition leads to a more complex state which is less dissipative, one displaying damped oscillations (so-called Lotka–Volterra behaviour). Kinetic stability and energy dissipation have evolved in opposite directions*.* Thus, through this simple kinetic simulation, one differing from natural selection between species variants (corresponding to concepts defined within the biological field), a more general view of evolution involving chemical autocatalysts is obtained. Once again we observe an instance in which the MEP principle is inapplicable, further reaffirming Ross’s critical MEP assessment [37]. The level of energy dissipation (corresponding to the amount of activated reactant **R** converted into inactivated products through the autocatalytic path *k*_2_) is influenced by contingent events, rather than by a general thermodynamic law. In fact, what the introduction of the predator into the system does do (leading to Lotka–Volterra oscillating behaviour, Figure 4), is to lead to an increase in the system’s complexity*.* This aspect will be discussed subsequently.

### Stability and complexity

Even though the Second Law drive towards equilibrium is brought about through the minimisation of the Gibbs free energy of the system, we learn from Figure 4 that the maximisation of free energy dissipation does not account for the direction of change. Indeed the system described in Figure 3 will never revert to its previous state in which **B** was absent and energy dissipation was higher. What the addition of **B** brings about is an increase in complexity, suggesting that it is not just stability/persistence which increases, and that whatever quantities are being optimised, they should also include a term related, whether directly or indirectly, to complexity. It is worth noting that the meaning of complexity considered here refers to the degree of organisation within the system, to the interconnections of its parts, and not just to the number and diversity of its components. This observation of increase in complexity supports the hypothesis that the evolution of reproducing systems is ruled by a Second Law analogue in which complexity plays a role similar to that of entropy during the evolution of non-replicative systems towards thermodynamic equilibrium [7,50–54]. Unfortunately, as complexity is notoriously difficult to both define and measure [59–61], quantification of such a Second Law analogue seems out of reach at present.

Thus though the evolution of a dynamic system based on entities able to self-reproduce is continuously governed by an increase of dynamic kinetic stability, predicting the result of long-term evolution becomes impossible, primarily because it depends on the particular path followed during the process. These complex systems can reach bifurcation points from which the system can evolve along different paths [10] rendering any prediction of evolutionary paths impossible. Evolutionary possibilities invariably depend on earlier choices. Additionally, the boundaries of a necessarily open system cannot be defined so that events in the environment can influence the future of the system. However, the impossibility of measuring dynamic kinetic stability is precisely the source of unlimited possibilities for evolution, its open-ended character coupled with its divergent nature [47]. Indeed, provided that the environment provides energy in sufficient quantities and potential to sustain life, there should be no end to the evolutionary process as neither DKS, nor the complexity which accompanies it, appear to have an upper bound.

### A free energy potential threshold as a requirement for the origin of life

**Figure 5:**
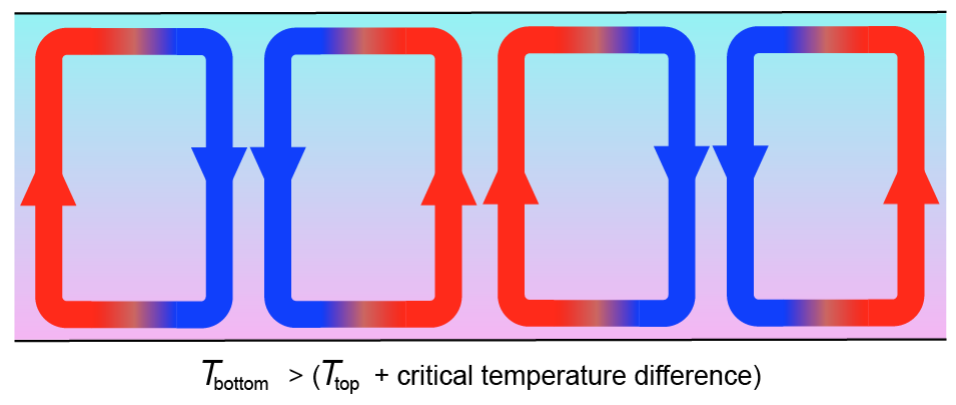
The Raleigh–Bénard instability. Convection takes place in a liquid layer provided that the temperature difference between the bottom and the top of the layer exceeds a threshold value.

Key conditions for observing physicochemical behaviour governed by dynamic kinetic stability is that the system is self-reproducing and able to undergo exponential growth [13–14,62–63]. These conditions further imply that the system is maintained in a far-from-equilibrium state and that the chemical autocatalytic process involved must be kinetically irreversible (i.e., the rate of the reverse reaction must be negligible on the timescale of reproduction/generation) [1,4]. The nature of this requirement may be understood more readily by analysing a well-known example of emergence of dissipative structures. One of the most studied is the emergence of convection when the bottom surface of a liquid layer is heated (Figure 5). It turns out that a low temperature gradient is insufficient for convection to be observed and the minimum gradient must exceed a threshold above which Raleigh–Bénard instability is observed (Figure 5). The result of convection is, of course, an increase of energy dissipation by the resulting non-linear process, though its emergence depends on the action of gravity and the laws of fluid dynamics.

Regarding the origin of life, we suggested that an analogous threshold is also present [4], which can be identified as a consequence for the need for kinetic irreversibility. Above that threshold (associated with a value of chemical free energy potential expressing a distance from equilibrium), kinetic selection among variants of autocatalysts becomes efficient [13–14,62–63], which reproduces similar behaviour to the one responsible for natural selection. The condition for irreversibility associated with this threshold, expressed as a reproduction/generation timescale shorter than that for the reverse process, has provided a means of semi-quantitatively assessing kinetic barriers [55–56]. This assessment was based on a relationship between time scale, kinetic barriers and temperatures, and taking into account the following hypotheses:

- a temperature as low as possible, but allowing the presence of water in its liquid state (higher temperature strongly increase the threshold),
- generation times of 1 second to 100 years.

The threshold can therefore be expressed as a minimum free energy potential corresponding to chemical quanta feeding the system in energy. Kinetic barriers needed for ensuring kinetic irreversibility correspond to a value of ca. 100 kJ mol^−1^ at 300 K. This value corresponds to a significant fraction of the free energy of covalent bonds (and then to the kinetic barriers commonly observed for their reactions), which is a strong indication that in a range of moderate temperatures (ca. 300 K), the chemistry of carbon – the element that most easily forms covalent bonds – should be preferentially involved in a self-organisation process based on the specificity of entities able to reproduce themselves. Moreover, the energy input allowing the irreversible formation of intermediates having a degree of activation equivalent to that of biochemical intermediates like ATP, requires a free energy potential exceeding a value of 150 kJ mol^−1^, equivalent to that of visible light [4,55–56]. Therefore, it turns out that considering the kinetic conditions for dynamic kinetic stability leads to the definition of conditions for the origin of life that more or less correspond to the conditions for the development of life on the primitive Earth (organic chemistry, liquid water and visible or UV light). Here again, some recent experimental work has shown how photochemistry could lead to biochemical building blocks compatible with further developments towards the origin of life [8–9].

### Evolvability and the origins of life

This discussion has not taken into account the ability of a system to evolve, which was not the goal of the present work, but is obviously a requirement for any possibility of evolution [64–65]. Extended possibilities for variation are indeed a requirement for systems to undergo open-ended evolution [66]. The storage of genetic information as a sequence in a polymer associated with template replication through base-pairing constitutes an efficient system to ensure evolvability. It is that evolvability which allows selection toward life as we know it on Earth. However, as the proximity from equilibrium has been mentioned above as a limitation, the higher affinity of long strands compared to fragments is the source of another limitation (product inhibition). That limitation, discovered for template replication by von Kiedrowski [67], leads to sub-exponential growth. It turns out, at least at this time, that no isolated system able to reproduce itself, presents all of the qualities required for the emergence of life: i.e., the replication of nucleic acids through base-pairing is limited by parabolic growth and autocatalytic networks present limited possibilities of variability. This situation has led many researchers in this field to support a co-evolutionary approach in which several sub-systems able to reproduce themselves could co-operate to initiate a possibility of natural selection [68–69]. It is worth noting that some years ago, the need for cooperation between sub-systems had already been suggested as a requirement for an autonomous self-reproducing system, through the pioneering work of Tibor Gánti [70]. If we consider that the process starting from inanimate matter to living organisms progressed through stages of increasing DKS, then the most important transitions very likely corresponded to the initiation of cooperative associations corresponding to both an increase in complexity of the system and its dynamic kinetic stability. The ground-breaking endosymbiotic theory put forward by Lynn Margulis [71] half a century ago to explain eukaryotic cell formation is in fact just a particularly striking example of a cooperative association in action. It is also important to emphasise that cooperation may either have involved a physical linkage between different components through direct binding or encapsulation, but that functional linkages in which reactants or products could be common to different systems would have been important as well.

### Organic chemistry and the origin of life

The lines of thought developed here point towards a global approach to account for the emergence of life as a consequence of contingent events that occur in a context in which kinetic driving forces towards more efficient self-reproducing systems are constrained by thermodynamics, as well as by the properties of covalent bonds involving carbon. They support the essential role of organic chemistry in the origin of life process as a result of the kinetic barriers associated with covalent bonds. It is encouraging that recent experiments have demonstrated that complex kinetic behaviour can be observed in simple organics [72–73], and is not particular to inorganic systems or enzymatic reaction networks. Our approach, beginning with the hypothesis of an auto-organisational process based on the kinetic properties of self-reproducing entities, leads to a semi-quantitative assessment of the environmental conditions required for a self-organisation process based on organic chemistry. It is instructive to note that this assessment is compatible with visible light as an energy source as well as moderate temperatures, both of which could be found at the surface of the early Earth. However, these considerations by themselves do not solve the question of the origin of life, or at least the point of initiation of an evolutionary process driven by an increase in DKS. The precise nature of the chemical species involved in that process remains unknown. Interestingly, however, recent investigations [74–75] have demonstrated that some kind of selective chemistry can simultaneously yield, via photochemical pathways, a wide range of precursors similar to those found in biochemistry (amino acids, nucleotides and lipid precursors).

## Conclusion

This paper attempts to place life and its emergence within a general physicochemical context. Once it is appreciated that life emerged from inanimate beginnings in a well-defined process with an identifiable driving force, the Chinese wall that has somehow managed to separate the conceptual worlds of animate and inanimate, can finally be breached. The biological and physical worlds are intimately connected through process. There is a process, explicit and physicochemically defined, that under appropriate contingent conditions, leads from chemistry to biology such that these two worlds merge into one. So, though life is a complex chemical system exhibiting complex kinetic behaviour, that complex behaviour can be traced back to self-reproducing chemical systems maintained far from equilibrium and directed by kinetic driving forces. Chemical systems able to evolve in the direction of increased dynamic kinetic stability – toward life – need to be endowed with three essential properties. They must be able to reproduce themselves, their structure should be compatible with the possibility of variation, and they should be maintained in a dynamic far from equilibrium state through a continual energy supply. Selection is then the inevitable consequence. According to Darwinian theory, it is selection that drives evolution. However, natural selection is a very specific process which applies to only a part of the natural world, and is seemingly detached from traditional physicochemical behaviour. Neither the distance from equilibrium nor the maximisation of energy dissipation constitute driving forces for the emergence of life but they correspond to a condition for its development for the former and a manifestation associated with their behaviour for the latter. The actual driving force for life is associated with the power of exponential growth that is expressed by self-reproducing entities. Moreover, the hypothesis of an auto-organisational process based on the kinetic properties of these entities leads to a semiquantitative assessment of the environmental conditions required for a self-organisation process, one based on established organic chemical processes. It is intriguing to note that this assessment is compatible with visible light as an energy source, and a moderate temperature, both of which would have been found on the surface of the early Earth.

This approach to biological systems that focuses on their emergence from chemical ones has some far reaching consequences. The “autonomy of biology” view of life [76], still deeply engrained within life science thinking, needs to be reassessed as it undermines attempts to understand biology’s deeper essence. The very fact that chemistry almost certainly evolved over time into biology is the clearest statement that the physical and biological worlds are merely two regions of a physicochemical–biological continuum. It also means that biological understanding in its deeper sense must lie in physics and chemistry. The awkward reality for biologists – that biology’s essence, secreted within those physicochemical origins lies largely outside the subject that purports to study it.

Finally, understanding life as a complex kinetic process allows conclusions to be drawn regarding the widely held view that life, its emergence and evolution, can be understood as a thermodynamic phenomenon. We believe that there is now clear evidence that argues against that thermodynamic viewpoint (though life processes are necessarily bound by thermodynamic constraints). The key points in support of a kinetic paradigm may be summarised as follows:

1. The cell, the fundamental unit of biology, has evolved from simpler chemical beginnings to minimise energy dissipation, not to maximise it. This is reflected in the extraordinary efficiency of the cell-reproduction apparatus which has evolved to maximise reproduction, not energy dissipation.

2. Whereas an evolutionary process toward increasing complexity is a widely observed phenomenon, the transition to that more complex state may lead to a reduction in energy dissipation, as expressed in a variety of experimental situations [37] as well as in the kinetic simulation described in this paper. The existence of clear exceptions to the energy dissipation view of life questions the validity of a general thermodynamic paradigm.

3. Kinetic pathways cannot, as a general rule, be deduced from thermodynamic factors. Any two thermodynamic states are potentially connected by an infinite number of kinetic pathways and extra-thermodynamic information is required to deduce which pathway is followed in any particular case. Given that all persistent replicative systems are in essence kinetic steady states, the evolutionary process based on that replicative essence must therefore also be kinetic in nature. Accordingly, any process governed primarily by kinetic factors is unlikely to be generally describable in thermodynamic terms.

A closing comment: in order to address the most general of life questions – for example, could life be based on an alternative chemistry, how could we identify such life forms – a more chemically explicit understanding of what life is, is necessary. Richard Feynman’s famous aphorism: “what I cannot create, I do not understand” points the way forward. Given the precise physicochemical description of the life process presented here and in earlier publications, specific chemical steps toward the synthesis of simple protolife systems are now indicated [54]. This goal, if and when achieved, would go a long way toward answering the perennial ‘what is life’ question, as well as answering the ahistorical question, how was inanimate matter of whatever kind able to evolve into life.

## Supporting Information

File 1 Conditions for exponential growth and steady states calculated for the system of Figure 3.
