# Supplementary material for: How and why kinetics, thermodynamics, and chemistry induce the logic of biological evolution
Source: Beilstein J Org Chem. 2017 Apr 7;13:665–74. doi: 10.3762/bjoc.13.66 (PMC5389199; doi:10.3762/bjoc.13.66)
Supplement: File 1 — Conditions for exponential growth and steady states calculated for the system of Figure 3. [file Beilstein_J_Org_Chem-13-665-s001.pdf]

Supporting Information

for

How and why kinetics, thermodynamics, and chemistry induce the logic of biological evolution

Addy Pross[1,2] and Robert Pascal[*3]

Address: [1]Department of Chemistry, Ben Gurion University of the Negev, Be’er Sheva 84105, Israel, [2] NYU Shanghai, 1555 Century Avenue, Pudong New Area, Shanghai, 200122 China, and [3]Institut des Biomolécules Max Mousseron, UMR5247 CNRS-University of Montpellier-ENSCM, CC17006, Place E. Bataillon, Montpellier F-34095, France

Email: Robert Pascal - robert.pascal@umontpellier.fr

*Corresponding author

Conditions for exponential growth and steady states calculated for the system of Figure 3

(1) In the absence of autocatalyst A:

When the system reaches a steady state:

$d[R]/dt = k_0 - k_1 [R]_{*1} = 0$

The steady state concentration of [R] can be assessed as:

$[R]_{*1} = k_0 / k_1$

(2) Autocatalyst A present:

Condition for growth of **A**:

$d[A]/dt = k_2 [A] [R] - k_3 [A] > 0$

The observation of an autocatalytic behaviour from **A** therefore requires the condition

$k_2 [R] > k_3$

The determination of steady states for R and A requires considering the following equations:

$d[R]/dt = k_0 - k_2 [A]_{*2} [R]_{*2} - k_1 [R]_{*2} = 0$

and

$d[A]/dt = k_2 [A]_{*2} [R]_{*2} - k_3 [A]_{*2} = 0$

Then either $[A]_{*2} = 0$

or $[R]_{*2} = k_3 / k_2$

and $k_0 - k_3 \times [A]_{*2} - k_1 \times k_3 / k_2 = 0$ and then $[A]_{*2} = (k_0 - k_1 \times k_3 / k_2) / k_3$

(3) Autocatalyst A and parasite B present:

Condition for growth of B:

$d[B]/dt = k_4 [A] [B] - k_5 [B] > 0$

so that $k_4 [A] > k_5$

Steady states:

$d[R]/dt = k_0 - k_2 [A]_{*3} [R]_{*3} - k_1 [R]_{*3} = 0$

$d[A]/dt = k_2 [A]_{*3} [R]_{*3} - k_3 [A]_{*3} - k_4 [B]_{*3} [A]_{*3} = 0$

$d[B]/dt = k_4 [A]_{*3} [B]_{*3} - k_5 [B]_{*3} = 0$

Which leads to either $[B]_{*3} = 0$,

or $[A]_{*3} = k_5 / k_4$ and $[R]_{*3} = k_0 / (k_1 + k_2 k_5 / k_4)$
